# Radiation-driven lipid accumulation and dendritic cell dysfunction in cancer

**DOI:** 10.1038/srep09613

**Published:** 2015-04-29

**Authors:** Fu Gao, Cong Liu, Jiaming Guo, Weimin Sun, Linfeng Xian, Dongchen Bai, Hu Liu, Ying Cheng, Bailong Li, Jianguo Cui, Chaoxiong Zhang, Jianming Cai

**Affiliations:** 1 Department of Radiation Medicine, Faculty of Naval Medicine, Second Military Medical University, Shanghai 200433, PR China; 2 National Key Laboratory of Medical Immunology & Institute of Immunology, Second Military Medical University, Shanghai 200433, China; 3 Department of Centre for Disease Prevention and Control, Chengdu Military Region, Chengdu610021, China

## Abstract

Dendritic cells (DCs) play important roles in the initiation and maintenance of the immune response. The dysfunction of DCs contributes to tumor evasion and growth. Here we report our findings on the dysfunction of DCs in radiation-induced thymic lymphomas, and the up-regulation of the expression of the *lipoprotein lipase (LPL)* and the *fatty acid binding protein (FABP4)*, and the level of triacylglycerol (TAG) in serum after total body irradiation, which contribute to DCs lipid accumulation. DCs with high lipid content showed low expression of co-stimulatory molecules and DCs-related cytokines, and were not able to effectively stimulate allogeneic T cells. Normalization of lipid abundance in DCs with an inhibitor of acetyl-CoA carboxylase restored the function of DCs. A high-fat diet promoted radiation-induced thymic lymphoma growth. In all, our study shows that dysfunction of DCs in radiation-induced thymic lymphomas was due to lipid accumulation and may represent a new mechanism in radiation-induced carcinogenesis.

Exposure to ionizing radiation has been implicated in cancer initiation and the promotion of cancer progression^1^,^2^,^3^. Radiation-induced mouse thymic lymphomas are among the classic models of radiation carcinogenesis. Many factors, like the Ras oncogene and p53 tumor suppressor gene, are involved in radiation-induced carcinogenesis^1^. We have previously shown that the activated ERK1/2 pathway may play a role, without involving STAT3, in the pathogenesis of γ-ray-induced thymic lymphomas in BALB/c mice^4^, and also that TLR4-Knockout mice were protected from radiation-induced thymic lymphomas by down-regulation of IL-6 and miR-21^5^.

Dendritic cells (DCs) specialize in acquiring, processing and presenting antigen to T cells, and are critical for antitumor immune response^6^,^7^,^8^,^9^. However, when DCs are defective, this can contribute to tumor evasion and growth^10^,^11^, which are processes mediated by various tumor-derived factors^10^,^12^. To investigate the pathogenesis of radiation-induced thymic lymphomas, gene expression in thymic lymphomas was analyzed. We observed low expression of genes associated with functional molecular marker genes in DCs and high expression of *Lipoprotein lipase* (*LPL*) and *fatty acid binding protein 4* (*FABP4*) genes. LPL has been shown to increase the uptake of lipid, and humans with *LPL* deficiency develop severe hypertriglyceridemia^13^, FABP play roles in fatty acid uptake, transport, and metabolism^14^.

A previous study has proven that increased uptake of extracellular lipids causes lipid accumulation in DCs, which in turn reduces their ability to effectively stimulate T cells^15^. Additionally, the adverse effect of dietary lipids intake on DCs functions has been confirmed by many studies^16^,^17^,^18^,^19^,^20^. These findings prompted us to investigate the potential role of lipid accumulation and DCs function in radiation-induced thymic lymphomas.

In this study we hypothesize that dysfunction of lipid metabolism leads to lipid accumulation in DCs, which in turn impairs the function of DCs and promotes thymic lymphomas growth. Our results support this hypothesis and may also provide a new mechanism of radiation-induced carcinogenesis cancer via lipid accumulation.

## Methods

### Mice and treatments

C57BL/6 mice, 5–6 weeks of age, and DO11.10 OVA_323–339_-specific TCR-transgenic mice with C57BL/6 background were purchased from the Chinese Academy of Sciences (Shanghai, China). All mice were housed in a Specific Pathogen-Free (SPF) facility for all experiments. All animal experiments were undertaken in accordance with the National Institute of Health “Guide for the Care and Use of Laboratory Animals” (NIH Publication No. 85-23, National Academy Press, Washington, DC, revised 1996), with the approval of the Laboratory Animal Center of the Second Military Medical University, Shanghai. All efforts were made to minimize the number of animals used as well as any suffering.

### Reagents

Recombinant mouse GM-CSF, IL-4, and ELISA kit for murine IL-12p40, IL-1β, IFN-γ, TGF-β, M-CSF, G-CSF, VEGF, GM-CSF, PGE_2_ and IL-6 were purchased from R&D Systems. Fluorescein-conjugated mAbs to Ia, CD86, CD80, CCR7 and isotype control mAbs, and BODIPY 493/503 were purchased from BD Pharmingen. Microbead-conjugated mAbs to CD4, CD11c were purchased from Miltenyi Biotec. Neutralizing Abs to IL-6 and TGF-β and isotype controls were purchased from R&D Systems. Acetyl-CoA carboxylase, 5-(tetradecycloxy)-2-furoic acid were purchased from Abcam. High fat diet and low fat diet^21^ were purchased from Shengong (Shanghai company).

### Total-body irradiation

A 60Co irradiator was used for total-body ionizing irradiation. Un-anaesthetized mice were placed in well-ventilated plastic boxes and exposed to the 60Co-γ radiation at a distance of 3 m 4 from the source. Four weekly sub-lethal doses of 1.75 Gy gamma-ray irradiation were delivered at a dose rate of 0.58 Gy/min as described previously^22^,^23^,^24^. The mice were then removed from the plastic box and allowed free access to food and drinking water. To evaluate lymphoma incidence, Three weeks after 6 Gy gamma-ray exposures, thymus were isolated from C57BL/6 mice. The thymus was inspected, and the number of mice with lymphoma were recorded, and the lymphoma incidence was calculated.

### RNA extraction and Real time q-PCR

RNA was extracted with Trizol reagent (Invitrogen, Carlsbad, CA, USA) according to the manufacturer's protocol. The cDNA synthesis and real-time qPCR were subsequently performed using the Qiagen system as described detail in previous studies^25^. The primers used are listed in Supplementary Table S1.

### Cells purification and preparation

Three weeks after 6 Gy gamma-ray exposures, thymus and spleen were isolated from C57BL/6 mice. Single cells were prepared by mechanical disruption and red cells depletion. These cells were collected separately and purified by anti-CD11c microbeads as DCs.

### Assays for Ag-specific CD4+ T cell response

For assay of Ag-specific CD4+ T cell proliferation splenic CD4+ T cells from DO11.10 OVA_323–339_-specific TCR-transgenic mice were positively selected with anti-CD4-coated microbeads (Miltenyi Biotec) by MACS and the cocultured with DCs treated as indicated in the presence of OVA_323–339_ peptide at a ratio of 1:10 (DC:T) in round-bottom 96-well plates (1 × 10^5^ T cells/200 μl/well) for 5 days. Proliferation of T cells was analyzed by double staining with anti-CD4+ and 7-AAD- cells were counted by FACS.

### Serum preparation and co-cultured with DCs

Whole blood was collected in test tubes by removing eyeball. 30 min later, the clot was removed by centrifuging at 2000 g for 10 min. The supernatant was collected as serum^26^. DCs were co-cultured with serum for 60 h (with or without TOFA), Then DCs were washed by PBS for further experiments.

### Preparation of DCs from mouse bone marrow

DCs were prepared from bone marrow progenitors according to a published method^27^, with minor modifications. Bone marrow mononuclear cells were prepared from mouse (5–6 weeks old) femur bone marrow suspensions by depletion of red cells and then were cultured at a density 2 × 10^6^ cells/ml in 6-well plates in RPMI 1640 medium supplemented with 10% FCS, 10 ng/ml of recombinant mouse granulocyte-monocyte colony-stimulating factor and 1 ng/ml of recombinant mouse IL-4. Nonadherent cells were gently washed out on day 4 of culture. At day 5, the dendritic proliferating clusters were collected and purified by anti-CD11c microbeads as immature DCs.

### Analysis of cell surface marker expression and cytokine intracellular staining

Fluorescein-conjugated monoclonal antibodies recognizing Ia, CD40, CD80, CD86, CCR7, and the respective isotype controls were purchased from BD-PharMingen. Before fluorescent antibody staining, all cells were incubated for 15 min at 4°C with antibody to CD16/CD32 at a concentration of 1 μg per 1 × 10^6^ cell per 100 μl and cells were incubated for a further 30 min at 4°C. The cells were washed once with ice-cold PBS, pH 7.2, containing 0.1 NaN3 and 0.5% BSA and were resuspended in 300 μl PBS. Flow cytometry was done with a FACSCalibur and data were analyzed with CellQuest software (BD Biosciences). The phenotype of cells from thymus, spleen were analyzed by LSR II flow cytometry (BD Biosciences) as described previously^12^,^24^. In details, the CD11c^+^ phenotype cells was treated as DCs. Cells were double-staining with CD11c-Ab and other molecular-Ab, then in the gate of CD11c positive, the other molecular expressions were assayed. IL-12 in cells assay: brefildin A was added for 7 h, then fixed by 4% paraformaldehyde for 30 min, then perforated by 0.1% saponin for 30 min, and co-cultured with mAb-IL-12 conjugated with FITC for 1 h, 4°C. The level of IL-12 in cells was assayed by FACS.

### Assay for cytokines, TAG, TC and Glucose

Cytokine in the supernatant of the DC-lipid system were assayed with ELISA kits (R&D System). Concentration of triacylglycerol (TAG), total cholesterol (TC) and glucose were assayed by Laboratory Medicine department of Changhai hospital, The Second Military Medical University. TAG, TC and glucose were assayed by Triglyceride Quantification Kit (ab65336), Cholesterol Quantification Kit (ab65359), Glucose Assay Kit (ab65333) separately (Abcam, Cambridge, UK).

### Lipid content analysis

To analyze the lipid content in cells, the lipophilic fluorescent dye BODIPY 493/503 was used. BODIPY 493/503 dye is bright, green fluorescent dye with similar excitation and emission to fluorescein (FITC). Cell were then washed and resuspended in 500 μl of BODIPY 493/503 at 0.5 μg/ml in PBS. Cells were stained for 15 min at 20°C before the analysis. All experiments with BODIPY performed on LSRII.

### Statistical Analysis

Data were presented as the mean ± s.d. from at least three independent experiments. The difference between groups were analyzed using two-tailed Student's *t* test when only two groups were compared. The difference between groups were analyzed using ANOVA when three or more than three groups were compared. Correlation analysis was performed by two-tailed Person's correlation coefficient analysis. Mice survival was determined by Kaplan-Meier analysis. Statistical analyses were performed using SPSS software (version 17.0). P < 0.05 was considered significantly different.

## Results

### DCs dysfunction in radiation-induced thymic lymphomas

**Figure 1:**
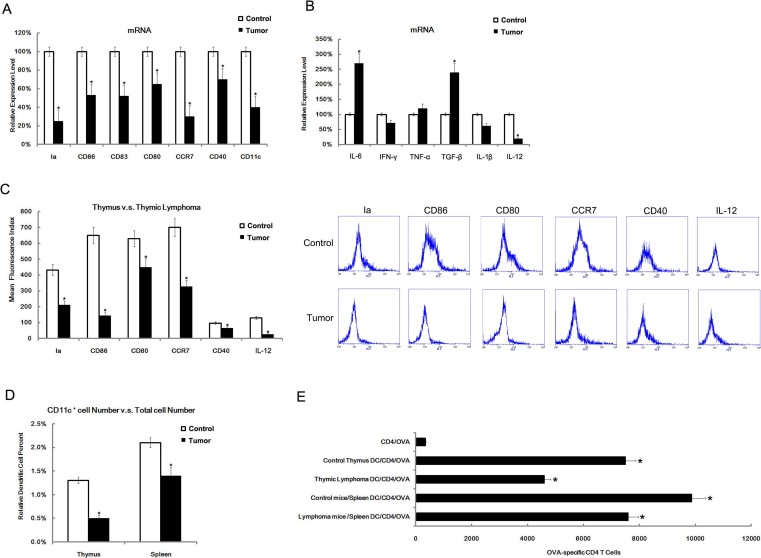
The phenotype, cytokine, number and T cell proliferation stimulating ability of DC in thymic lymphomas. Thymic lymphomas were isolated from 15 mice with radiation-induced thymic lymphomas. Total mRNA was then extracted for qRT-PCR analysis. Data were normalized to GAPDH. The expression in normal thymus tissues was arbitrarily defined as 100% (A). Cytokine levels were assayed by qRT-PCR. Data were normalized to GAPDH. The expression in normal thymus tissues were arbitrarily defined as 100%. (B). Thymic lymphomas were freshly isolated from C57BL/6 mice with thymic lymphomas. After acquiring single cells, these cells were double-stained with CD11c and co-stimulator or cytokine molecular FACS antibody. The mean fluorescence index of other molecules in the gate of CD11c positive was assayed. Normal thymic tissues were used as a control (C). After the preparation of single cell from the thymus or spleen, the percentage of CD11c positive cells was calculated (D). The CD4^+^ T cells from DO11.10 OVA_323–339_ specific (TCR-transgenic × C57BL/6) F hybrid mice were cocultured with DCs from thymic lymphomas or from the spleen of radiation- treated mice in the presence of OVA peptides. 5 days later, the total number of viable CD4^+^ (CD4^+^-7-AAD^−^) cells in each well was measured by FACS analysis (E). All data are presented as the mean ± s.d. of three separate experiments. *P < 0.05.

We compared the gene-expression profiles of radiation-induced thymic lymphomas and normal adjacent-matched thymus tissues by gene microarray. This comparison revealed that the expression of genes for co-stimulatory molecules and cytokines associated with DCs function in thymic lymphomas were down-regulated (data not shown). To confirm this finding, we analyzed the expression of co-stimulatory molecules genes and cytokines in thymic lymphomas and appropriate control tissues by qRT-PCR, and found lower levels of Ia, CD86, CD83, CD80, CCR-7, CD40 and CD11c in thymic lymphomas (Fig. 1A), and higher levels of IL-6, TGF-β, and lower level of IL-12(Fig. 1B). Furthermore, by gating of the CD11c^+^ DCs cell population using flow cytometry analysis, we confirmed that the Ia, CD86, CD80, CCR7, CD40 and IL-12 proteins of CD11^+^ DCs were all down-regulated in thymic lymphomas (Fig. 1C). Since the percentage of DCs in the thymus and spleen indicated that thymic lymphomas have a small percentage of DCs in the thymus and spleen (Fig. 1D), we decided to test function of DCs directly. Therefore, DCs in thymus and spleen were acquired by CD11c^+^ sorting and were then tested for their T cell stimulating ability in a T cell proliferation experiment. We found that DCs from thymus or spleen both showed reduced T cell- stimulating ability (Fig. 1E). These data confirmed the dysfunction of thymus and splenic DCs of mice with radiation-induced thymic lymphomas.

### Serum from thymic lymphomas mice induced the dysfunction of DCs

**Figure 2:**
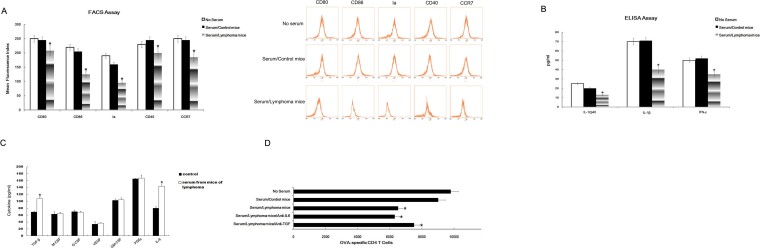
Serum from mice with thymic lymphomas induced the dysfunction of DCs. Immature DCs were co-cultured with serum from mice with radiation-induced thymic lymphomas mice for 60 h and then labeled with antibodies corresponding to CD80, CD86, Ia, CD40 and CCR7, for phenotypic analysis by FACS. Serum from WT mice was used as the control (A). ELISA was used to assay cytokines (IL-12p40, IL-1 and IFN-γ) in serum-pretreated DCs (B). ELISA was used to assay TGF-β, M-CSF, G-CSF, VEGF, GM-CSF, PGE_2_ and IL-6 in the serum of mice with radiation-induced thymic lymphomas(C). TGF-β neutralizing Ab (10 μg/ml) and IL-6 neutralizing Ab (10 μg/ml) were added at the beginning of serum/DC coculture. Isotype-matched Ab was included as a negative control. After 60 h, DCs were harvested and further cocultured with CD4^+^ T cells from DO11.10 OVA_323–339_-specific (TCR-transgenic × C57BL/6) F1 mice for 5 days in the presence of OVA, Finally, the number of viable CD4^+^ T cells (CD4^+^7-AAD^−^) were detected by FACS analysis (D). All data are presented as the mean ± s.d. of three separate experiments. *P < 0.05.

Previously we showed that immunosuppressive cytokines secreted by tumor cells can lead to dysfunction of DCs^12^. Thus we hypothesized that the immunosuppressive cytokines in the serum of radiation-induced thymic lymphomas mice may impair the functional ability of DCs. To test this hypothesis, we co-cultured DCs with serum from mice with radiation-induced thymic lymphomas for 60 h, and then examined the function and cytokines of DCs. We found decreased expression of several cytokines on the surface of DCs, including CD80, CD86, Ia, CD40 and CCR7 (Fig. 2A).

Additionally, expression was down-regulated for DCs secreted cytokines IL-12p40, IL-1 and IFN-γ (Fig. 2B). To identify the serum factors contributing to these effects, we measured the immunosuppressive cytokines level in serum from mice with radiation-induced thymic lymphomas, and found that the level of TGF-β and IL-6 were higher than in the control (Fig. 2C). Thus, it is possible that TGF-β and IL-6 in serum may be involved in the DCs dysfunction in thymic lymphoma. To confirm the role of TGF-β and IL-6, we performed a T cell proliferation experiment in the presence or absence of TGF-β and IL-6 neutralizing antibody in DCs-serum system separately, and found that only anti-TGF-β could partly restore the T cell stimulating function of DCs (Fig. 2D). Accordingly, we concluded that another immunosuppressive factors may exist in the serum of mice with radiation-induced thymic lymphomas.

### Triacylglycerol up-regulation in serum and lipid accumulation in DCs

**Figure 3:**
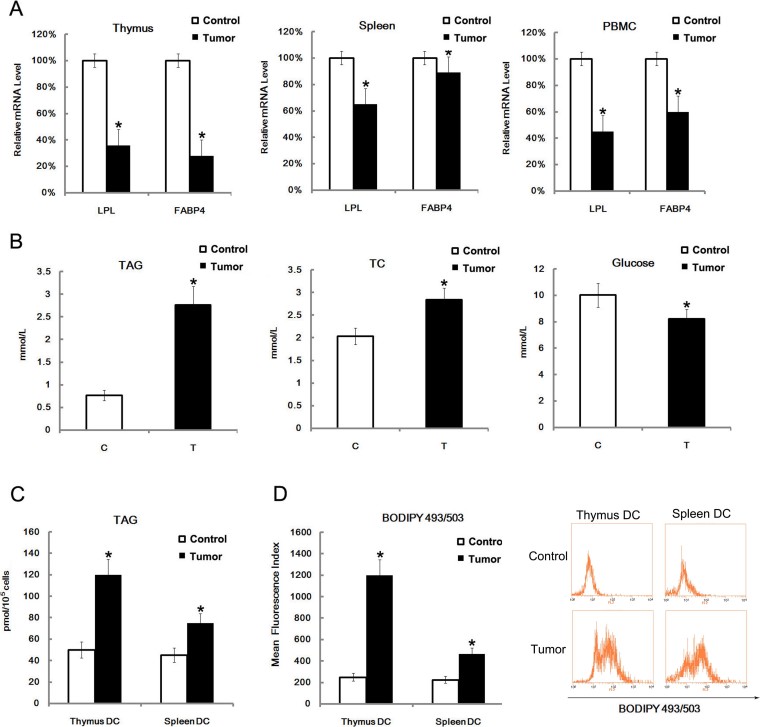
LPL and FABP4 levels were down-regulated, TAG was up-regulated and lipid content in DC was increased in radiation-induced thymic lymphomas. Thymus, spleen and PBMC were isolated from mice with radiation-induced thymic lymphomas. Expression of *LPL* and *FABP4* were assayed by qRT-PCR analysis of purified total mRNA (A). The TAG, TC and glucose levels in serum were assayed. Data are presented as the mean ± s.d. of three independent samples (B). The thymus and spleen were isolated, and thymic and splenic DCs were purified by sorting CD11c^+^ positive cells. The disrupted cells were then assayed for TAG level (C). Purified thymic and splenic DCs were stained with BODIPY493/503 (D). All data are presented as the mean ± s.d. of three independent experiments. *P < 0.05.

The results from microarray-base gene expression analysis showing the down-regulation of *LPL* and *FABP4* in mice with radiation-induced thymic lymphomas hinted at the involvement of these unknown immunosuppressive factors in thymic lymphomas pathogenesis. For this reason, qRT-PCR analysis was performed to measure expression in different cell types. The levels of *LPL* and *FABP4*, both of which are involved in lipid uptake and metabolism, was down-regulated in the thymus, spleen and PBMC of mice with thymic lymphomas, compared to the levels in mice that did not receive radiation treatment (Fig. 3A). Next, we tested the level of triacylglycerol (TAG), total cholesterol (TC) and glucose in the serum of mice with thymic lymphomas, and found that TAG level in serum of thymic lymphomas mice was increased greatly (Fig. 3B). As a high level of TG could lead to lipid accumulation in DCs and their dysfunction^15^, we tested the TAG level in splenic and thymic DCs, and found that the TAG level in DCs from thymic lymphomas mice was higher than in control mice (Fig. 3C). In addition, the lipid content in DCs from mice with thymic lymphomas was also higher (Fig. 3D).

### Lipid accumulation led to the dysfunction of DCs

**Figure 4:**
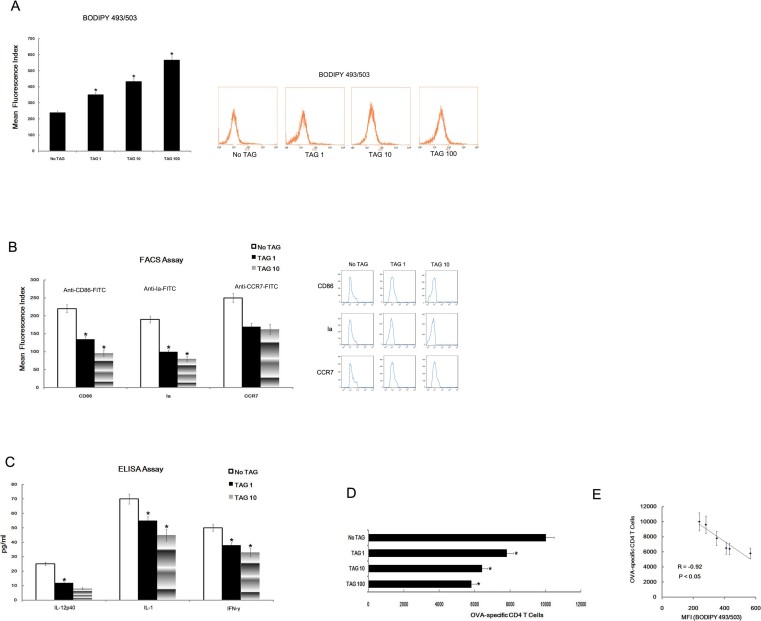
TAG led to lipid accumulation and dysfunction of DC. *In vitro* bone marrow progenitor-derived DCs were treated with TAG (1, 10 or 100 mmol/L) separately. Next, lipid content was analyzed in DCs by BODIPY493/503 staining (A). After pretreatment of DCs (1 or 10 mmol/L TAG co-cultured), the expression of CD86, Ia and CCR7 expression in DCs were analyzed by FACS analysis (B). After the pretreatment of DCs (1 or 10 mmol/L TAG co-cultured), DCs were washed by PBS 3 times, 24 h later, the supernatant of cells (6 × 10^5^ cells/well) was collected for ELISA assay (C). After pretreatment of DCs (1, 10 or 10 mmol/L TAG co-cultured), DCs were washed by PBS 3 times, harvested, and further co-cultured with CD4^+^ T cells from DO11.10 OVA_323–339_-specific (TCR-transgenic × C57BL/6) F1 mice for 5 days in the presence of OVA. Finally, the number of viable CD4^+^ T cells (CD4^+^7-AAD^−^) was detected by FACS (D). The correlation between the number of viable CD4^+^ T cells and the lipid content in DCs (BODIPY493/503 staining) was analyzed by two-tailed Person's correlation coefficient analysis (E). All data are presented as mean ± s.d. of three independent experiments. *P < 0.05.

A series of *in vitro* experiments were conducted to test whether lipid accumulation led to the dysfunction of DCs. We co-cultured DCs with different TAG concentrations, then measured lipid accumulation, DCs surface function molecular markers, cytokines levels, and T cell stimulating ability. The results indicate the increasing TAG led to lipid accumulation in DCs (Fig. 4A). Moreover, TAG could also down-regulate the expression of CD86 and Ia (Fig. 4B), and of IL-12p40, IL-1, IFN-γ level (Fig. 4C) in a concentration-dependent manner. Most importantly, TAG also reduced the T cell proliferation stimulating ability of DCs in a dose-dependent way (Fig. 4D), with a high correlation between the number of proliferating T cells and the lipid content of DCs (Fig. 4E).

### Inhibition of lipid accumulation restored the DC function

**Figure 5:**
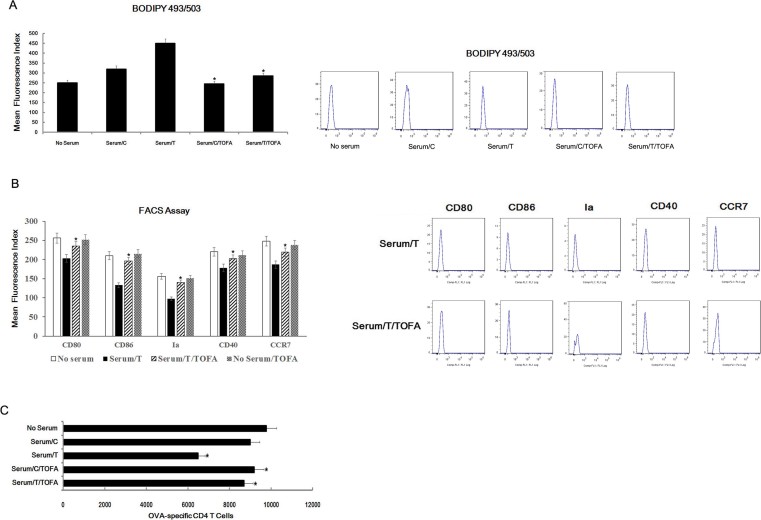
TOFA inhibited lipid accumulation and restored the DC function. In the presence of TOFA, immature DCs were co-cultured with serum from mice with radiation-induced thymic lymphomas for 60 h, DCs were stained with BODIPY493/503 for lipid content analysis (A). In the presence or absence of TOFA, the expression of CD80, CD86, Ia, CD40 and CCR7 of serum treated DCs was analyzed by FACS analysis (B). Immature DCs were cocultured with serum from mice with radiation-induced thymic lymphomas for 60 h in the presence of TOFA, harvested and then further co-cultured with CD4^+^ T cells from DO11.10 OVA_323–339_-specific (TCR-transgenic × C57BL/6) F1 mice for 5 days in the presence of OVA. Finally, the number of viable CD4^+^ T cells (CD4^+^7-AAD^−^) was detected by FACS analysis(C). All data are presented as the mean ± s.d. of three independent experiments. *P < 0.05.

To confirm the direct involvement of lipid accumulation in the dysfunction of DCs, rescue experiments were performed, To do this, fatty acid levels were regulated with an inhibitor of acetyl-CoA carboxylase, 5-(tetradecycloxy)-2-furoic acid (TOFA)^28^. Since TAG undergo rapid degradation in the cells, maintaining requires active fatty acid synthesis^15^. When synthesis is blocked, cells are unable to sustain high levels of triacylglycerol (Fig. 5A). FACS analysis showed that CD80, CD86, Ia and CCR7 expression was restored by TOFA treatment (Fig. 5B). Significantly, in the presence of serum of mice with thymic lymphomas, the treatment with TOFA considerably improved the ability of DCs to stimulate T cell proliferation (Fig. 5C). Hence, the rescue experiments results confirmed the role of lipid in DCs function.

### High fat dietary promoted radiation-induced thymic lymphomas growth

**Figure 6:**
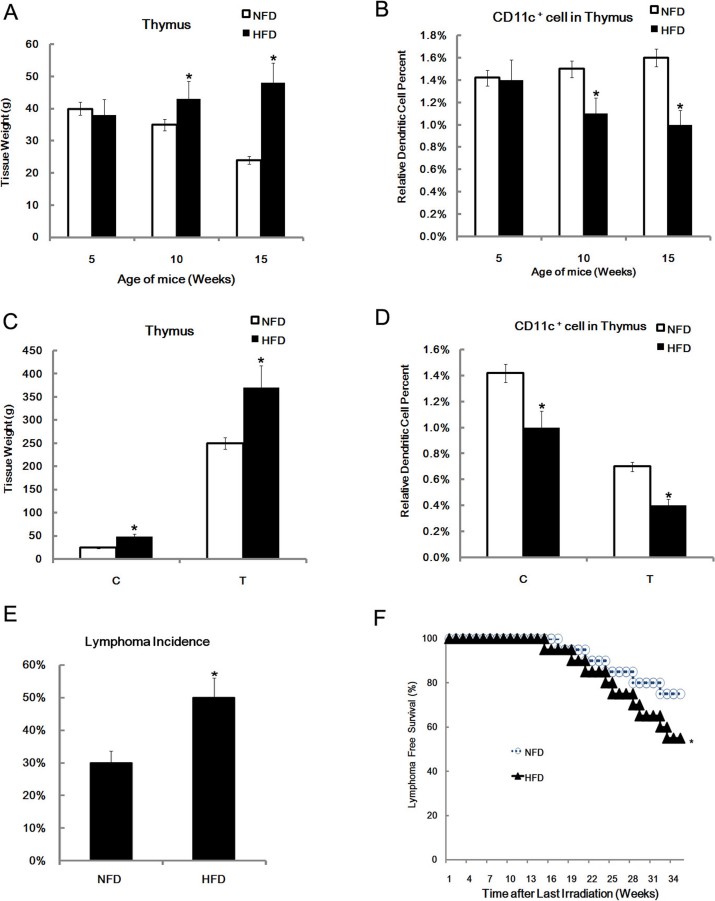
High-fat diet increased lymphoma incidence and reduced the survival rate of radiation-treated mice. Twenty C57B/L6 mice were fed a high fat diet (HFD) for 15 weeks, then the thymus was isolated and weighted, 20 mice fed with normal fat diet (NFD) served as the control (A). Single cells were prepared, and stained with Ab to CD11c. The CD11c positive cells were analyzed by FACS analysis (B). Twenty mice with radiation-induced thymic lymphomas mice were fed with the HFD or NFD, and the weight of the thymus was evaluated. 20 WT mice were used as the control (C). Twenty mice with radiation-induced thymic lymphomas were fed the HFD or NFD, and the percentage of DCs in thymus was examined by FACS analysis. Twenty WT were used as control (D). Twenty C57B/L6 mice were fed with the HFD for 15 weeks, the other 20 mice were fed the NFD as the control. In the last four weeks, mice received radiation treatment as described in the method. All mice were then euthanized to assess lymphoma incidence (E). The survival status of these treated mice were recorded after radiation treatment. The Kaplan-Meier curves were drawn according to the fat diet (F). All data are presented as the mean ± s.d. of three independent experiments. *P < 0.05.

The above results indicated that lipid accumulation led to dysfunction of DCs, which in turn promoted thymic lymphomas growth. Accordingly, we conjectured that a high fat dietary (HFD) enhanced radiation-induced thymic lymphomas by impairing the function of DCs. To explore this possibility, after radiation, we fed mice with high fat dietary or normal fat dietary (NFD) for 15 weeks, then thymus weight and DCs were measured. We found that HFD promoted thymic tissue growth (Fig. 6A), and decreased the percent of DCs in thymus (Fig. 6B). In mice with radiation-induced thymic lymphomas, the HFD increased thymus weight (Fig. 6C), and decreased the percentage of DCs in the thymus (Fig. 6D). Importantly, HFD increased the incidence of radiation-induced thymic lymphomas in mice (Fig. 6E), and survival analysis revealed that the HFD led to diminished lymphoma-free survival rate (Fig. 6F).

## Discussion

In this study, we primarily found dysfunction of DCs in radiation induced thymic lymphomas. Subsequently we found, *in vitro*, that the serum of mice with thymic lymphomas led to lipid accumulation in bone marrow-derived DCs and their dysfunction. The key factor in this process was proven to be TAG. In a previous study, lipid accumulation and dysfunction in DCs were also identified in lymphomas^15^. We presume that the accumulation of lipids might be due to increased synthesis of fatty acids or may result from increased lipid uptake from plasma. Radiation up-regulated the *LPL* and *FABP4* expression, and the high level of TAG in serum led to the lipid accumulation in DCs. Interestingly, in the previous study^15^, DCs from tumor-bearing mice showed preferential up-regulation of the macrophage scavenger receptor (Msr1, or CD204), and scavenger receptors represent a major route in the acquisition of fatty acids by DCs and macrophages^29^,^30^,^31^. In this work, the levels of Msr1 were not measured, but may similarly be up-regulated in DCs.

Lastly, we also found that HFD promoted radiation-induced thymic lymphomas growth. This finding is consistent with other studies^32^. Indeed, diet-induced obesity has many consequences including pathologies of diverse organ systems as well as cancers of the liver, kidney, and pancreas. In addition, our data highlight the role of HFD in radiation-induced carcinogenesis. Whether low fat diet has a radiation protective role is an important outstanding question.

We previously showed that HMGB1 was released from radiation-induced dying thymus cells. HMGB1 in turn activated TLR4 and elevated the pro-tumor factors IL-6 and miR-21, together with other important factors like MMP9 and miR-155, to induce carcinogenesis^5^. Here, using a similar radiation-induced thymic lymphoma model, we found the dysfunction of DCs, though HMGB1 could contribute to anticancer chemotherapy and radiotherapy via DCs^33^.

The context in radiation induced carcinogenesis is very complex and a number of factors and cells are involved. The discrepancy between studies may be due to analysis of different cross-sections data. DCs seem to be the key factor in radiation-induced carcinogenesis, and showed different roles different contexts.

In conclusion, we have confirmed the dysfunction of DCs in radiation-induced thymic lymphomas. Up-regulation of TAG in serum led to lipid accumulation and dysfunction in DCs. Our data highlight the role of DCs in radiation-induced thymic lymphomas and reveal a new mechanism of radiation-induced carcinogenesis.

## Author Contributions

F.G. and C.L.: study concept and design, carried out experiments. J.G., W.S., L.X., D.B., H.L., Y.C., B.L. and J.C.: carried out experiments. C.Z.: draft of the manuscript. J.C.: study design, obtained funding.

## Supplementary Material

Supplementary Information Supplementary information
