# Supplementary material for: Radiation-driven lipid accumulation and dendritic cell dysfunction in cancer
Source: Sci Rep. 2015 Apr 29;5:9613. doi: 10.1038/srep09613 (PMC4413852; doi:10.1038/srep09613)
Supplement: Supplementary Information [file srep09613-s1.pdf]

Radiation-driven lipid lipid Accumulation and dendritic cell dysfunction in cancer

Fu Gao[1#], Cong Liu[1#], Jiaming Guo[1#], Weimin Sun[2], Linfeng Xian[1], Dongchen Bai[1], Hu Liu[1], Ying Cheng[1], Bailong Li[1], Jianguo Cui[1], Chaoxiong Zhang[3*], Jianming Cai[1*]

[1]Department of Radiation Medicine, Faculty of Naval Medicine, Second Military Medical University, Shanghai 200433, PR China;

[2]National Key Laboratory of Medical Immunology& Institute of Immunology, Second Military Medical University, Shanghai 200433, China

[3]Department of Centre for Disease Prevention and Control, Chengdu Military Region, Chengdu610021, China

[#]These authors contributed equally to this paper.

*Correspondence: Chaoxiong Zhang & Jianming Cai. Address:

Department of Centre for Disease Prevention and Control, Chengdu Military Region, 12, Tianxian Road 610021， Chengdu; P.R. China Fax: +86-028-82871362. E-mail: zcx7792@126.com (Chaoxiong Zhang)

Department of Radiation Medicine, Second Military Medical University; 800, Xiangyin Road 200433, Shanghai; P.R. China Fax: +86-21-81871149. E-mail: caijianming882003@163.com (Jianming Cai).

Supplementary Table 1 Primers used

Ia F	GTGGTGCTGATGGTGCTG
Ia R	CCATGAACTGGTACACGAAATG
CD80 F	GGCAAGGCAGCAATACCTTA
CD80 R	CTCTTTGTGCTGCTGATTCG
CD83 F	CCAGCCGCATGTCGCA
CD83 R	CGAGCTGCAGATGGTAGTGT
CD86 F	TCTCCACGGAAACAGCATCT
CD86 R	CTTACGGAAGCACCCATGAT
CCR7 F	GAGGAAAAGGATGTCTGCCACG
CCR7 R	GGCTCTCCTTGTCATTTTCCAG
CD40 F	CTGTTTGCCATCCTCTTGGT
CD40 R	CGACTCTCTTTGCCATCCTC
CD11c F	CTGGATAGCCTTTCTTCTGCTG
CD11c R	GCACACTGTGTCCGAACTC
IL-6 F	CCGGAGAGGAGACTTCACAG
IL-6 R	GGAAATTGGGGTAGGAAGGA
IFN-γF	TGACCAGAGCATCCAAAAGA
IFN-γR	CTCTTCGACCTCGAAACAGC
TNF-α F	TACTGAACTTCGGGGTGATTGGTC
TNF-α R	CAGCCTTGTCCCTTGAAGAGAACC
TGF-β F	CACCGGAGAGCCCTGGATA
TGF-β R	TGTACAGCTGCCGCACACA
IL-1βF	AAA TGC CTC GTG CTG TCT GAC C
IL-1βR	CTG CTT GAG AGG TGC TGA TGT ACC
IL-12 F	CCACTCACATCTGCTGCTCCACAAG
IL-12 R	ACTTCTCATAGTCCCTTTGGTCCAG
LPL F	AGGCATACAGGTGCAACTCC
LPL R	TGTCCAGTGTCAGCCAGACT
FABP4 F	ACTGGGCCAGGAATTTGACG
FABP4 R	CTCGTGGAAGTGACGCCTT
